# Validation of Igls Criteria for Islet Transplant Functional Status Using Person-Reported Outcome Measures in a Cross-Sectional Study

**DOI:** 10.3389/ti.2023.11659

**Published:** 2023-09-26

**Authors:** Zoe Bond, Saffron Malik, Ayat Bashir, Rachel Stocker, Jocelyn Buckingham, Jane Speight, James A. M. Shaw

**Affiliations:** ^1^ Translational and Clinical Research Institute, Newcastle University, Newcastle upon Tyne, United Kingdom; ^2^ School of Biomedical, Nutritional and Sport Sciences, Newcastle University, Newcastle upon Tyne, United Kingdom; ^3^ Newcastle upon Tyne Hospitals NHS Foundation Trust, Newcastle upon Tyne, United Kingdom; ^4^ School of Psychology, Institute for Health Transformation, Deakin University, Geelong, VIC, Australia; ^5^ The Australian Centre for Behavioural Research in Diabetes, Diabetes Victoria, Carlton, VIC, Australia

**Keywords:** hypoglycaemia, Igls, PROMs, islet, transplant

## Abstract

Associations between islet graft function and well-being in islet transplant recipients requiring exogenous insulin remain unclear. This cross-sectional analysis compared person-reported outcome measures in 15 adults with type 1 diabetes whose islet transplants were classified according to Igls criteria as “Good” (*n* = 5), “Marginal” (*n* = 4) and “Failed” (*n* = 6) graft function. At a mean of 6.2 years post-first islet transplant, 90% reduction in severe hypoglycaemia was maintained in all groups, with HbA1c (mean ± SD mmol/mol) 49 ± 4 in recipients with “Good” function; 56 ± 5 (“Marginal”); and 69 ± 25 (“Failed”). Self-reported impaired awareness of hypoglycaemia persisted in all groups but those with “Good” function were more likely to experience symptoms during hypoglycaemia. “Marginal” function was associated with greater fear of hypoglycaemia (HFS-II score: “Marginal”: 113 [95, 119]; “Failed”: 63 [42, 93] (*p* = 0.082); “Good”: 33 [29, 61]) and severe anxiety (GAD7: “Marginal”): 21 [17, 21]; “Failed”: 6 [6, 6] “Good”: 6 [3, 11]; (*p* = 0.079)), diabetes distress and low mood. Despite clear evidence of ongoing clinical benefit, Igls criteria ‘Marginal’ function is associated with sub-optimal well-being, including greater fear of hypoglycaemia and severe anxiety. This study provides person-reported validation that “Good” and “Marginal” graft function are differentiated by general and diabetes-specific subjective well-being, suggesting those with “Marginal” function may benefit from further intervention, including re-transplantation.

## Graphical Abstract

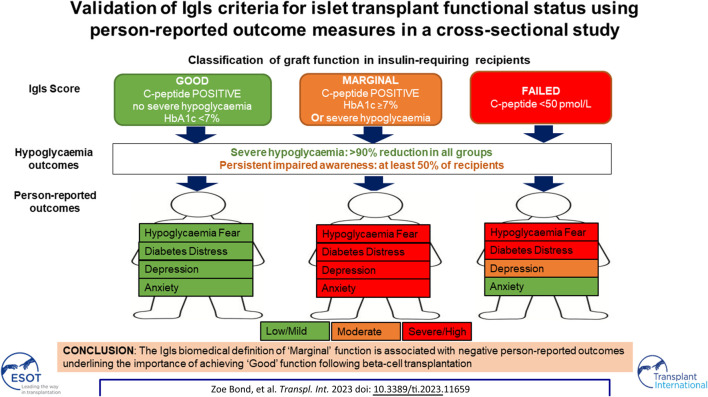

## Introduction

Following seminal success in Edmonton [1], intraportal transplantation of deceased donor isolated pancreatic islets has become established as standard-of-care for selected individuals with type 1 diabetes in healthcare systems around the world [2]. A National Health Service (NHS) funded integrated programme for islet transplant was commissioned in the United Kingdom in 2008 to provide equitable access to adults with C-peptide negative type 1 diabetes complicated by life-threatening hypoglycaemia despite optimal conventional medical management.

The goal of the NHS programme was to prevent further severe hypoglycaemia without the expectation of insulin independence. At the outset, the National Institute for Health and Care Excellence identified core audit criteria as confirmation of graft function through C-peptide positivity; reduction in numbers of severe hypoglycaemic events; attainment of HbA1c less than 53 mmol/mol (7%); and reduction in exogenous insulin dose [3]. In 2017, the International Pancreas & Islet Transplant Association (IPITA) and the European Pancreas & Islet Transplant Association (EPITA) proposed the Igls criteria using these outcome measures to define islet graft status. “Optimal function” necessitated insulin independence and a consensus was reached around definitions of “Good” and “Marginal” graft function [4, 5].

In the absence of insulin independence, associations between level of islet graft function and overall health status/well-being remain unclear. Validation of the Igls classification using patient-reported outcome measures (PROMs) was advocated in the original consensus statement but has not previously been undertaken [4]. We aimed to examine associations between Igls criteria and person-reported hypoglycaemia awareness; behaviours and fears around low and high glucose levels; diabetes distress; and anxiety/depressive symptoms in a cross-sectional study of previous islet transplant recipients, with an ongoing requirement for self-administered insulin therapy, at a single UK centre.

## Methods

### Study Design

The study was conducted between April and June 2022 following ethical approval (REC number 07/Q0904/11) to recruit participants who had received one or more percutaneous, transhepatic, intra-portal deceased donor pancreatic islet infusions at the Newcastle upon Tyne Hospitals NHS Foundation Trust within the NHS islet transplant programme. Inclusion criteria included ≥2 episodes of severe hypoglycaemia requiring assistance in treatment [6] over the 2 years before first islet transplant, with pre-transplant meal tolerance test stimulated C-peptide of <50 pmol/L and current requirement for exogenous insulin. In this cross-sectional study primarily designed to interrogate the Igls criteria proposed “boundary” between those with “good” and “marginal” islet graft function, we agreed *a priori* to exclude recipients with insulin independent “optimal” function.

A questionnaire pack was compiled for participant completion to assess hypoglycaemic episodes and impaired awareness; attitudes and behaviours towards hyper- and hypoglycaemia; diabetes-associated distress and problems; and anxiety/depression (Supplementary Table S1). Instruments which have previously been established as acceptable to, and validated in, adults with established type 1 diabetes were selected through a consensus reached by a consultant diabetologist, a diabetes clinical research fellow with experience in qualitative data collection, a health psychologist and a clinical psychologist. Acceptability, understandability, utility and face validity were confirmed in previous islet transplant recipients before finalisation. In keeping with published scoring systems, it was agreed that missing items would be replaced by the mean score of the non-missing items where less than 20% of items were missing in the Attitudes to Awareness of Hypoglycaemia (A2A) [7], Problem Areas in Diabetes (PAID) [8] (PAID), Hospital Anxiety and Depression Scale (HADS) [9], and 9-item Patient Health Questionnaire (PHQ-9) [10]; or where less than 25% of items were missing in Hypoglycaemia Fear-Survey-II (HFS-II) [11] and Hyperglycaemia Avoidance Scale (HAS) [12]. The Gold score [13], 7-item Generalised Anxiety Disorder Scale (GAD-7) [14] and Type 1 Diabetes Distress Score (T1DDS) [15] scortes were included when completed without missing items. The Hypoglycaemia Awareness Questionnaire (HypoA-Q) [16] was completed in long-form with novel analysis of “Symptom Frequency” and “Symptom Level” subscales. Participants were asked to comment on the utility and acceptability of each questionnaire in addition to any preference for particular measures.

In parallel with questionnaire completion, demographics, transplant history and biomedical data enabling Igls graft status classification according to published criteria [5] were obtained from participants’ most recent follow-up visit recorded on electronic healthcare records. C-peptide samples were analysed in a central reference laboratory by Siemens Immulite 2000 assay (Erlangen, Germany).

Statistical analysis was completed using SPSS statistics software version 28.0. Data normality was determined by Shapiro-Wilks test with age, diabetes duration and other parametric data presented as mean ± standard deviation and non-parametric data as median [quartile 1, quartile 3]. Categorical data are shown as number (%). Means were compared using one-way ANOVA with post-hoc testing using Tukey’s test. Medians were compared using Kruskal-Wallis test and categorical variables using Fisher’s exact test. *p* < 0.05 was considered statistically significant.

## Results

**FIGURE 1:**
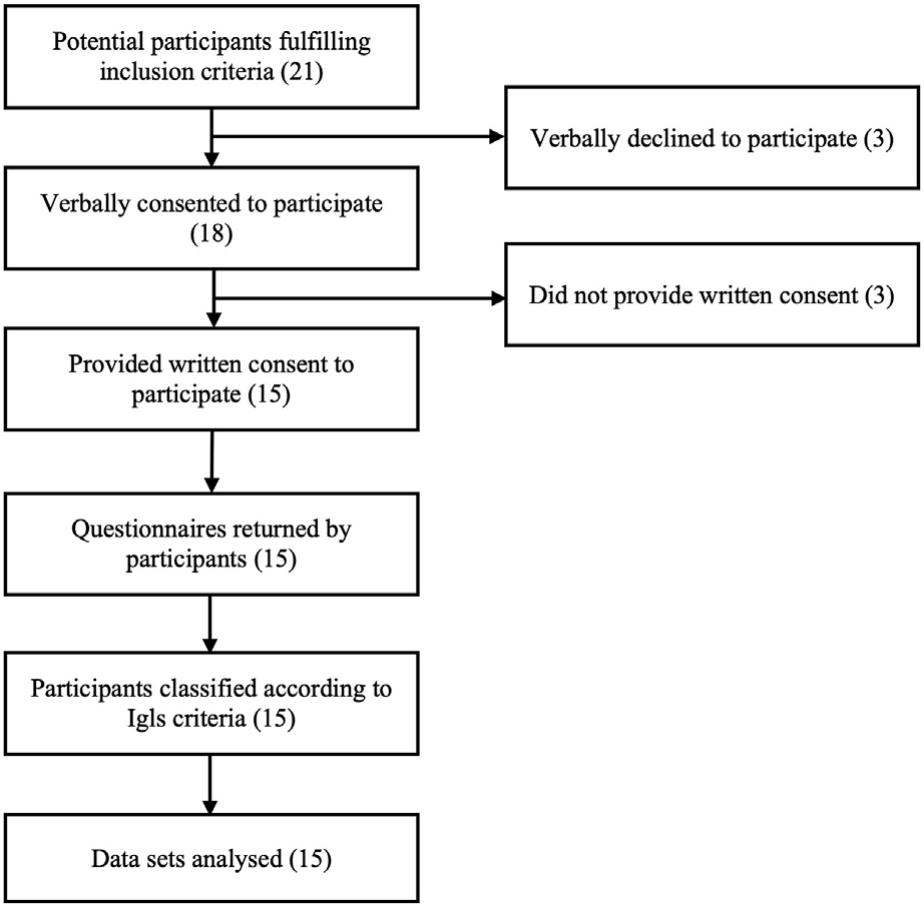
CONSORT diagram.

Twenty-one islet transplant recipients fulfilling inclusion criteria were approached for potential participation and 15 returned questionnaires following written informed consent (Figure 1). In participants with ongoing graft function, biomedical data were retrospectively collected from a follow-up visit within 6 months of questionnaire completion, with the exception of a single participant providing questionnaire data during pregnancy in whom pre-conception biomedical data were used. In participants with graft failure, biomedical data including C-peptide <50 pmol/L were collected from a single visit which may have preceded questionnaire completion by >6 months (but with confirmed clinical stability between biomedical and patient-reported data collection).

All participants had type 1 diabetes (absolute C-peptide negativity confirmed by pre-transplant meal tolerance test) complicated by recurrent severe hypoglycaemia requiring assistance with treatment, with 52 ± 98 events over the 12 months prior to transplantation. Twelve (80%) were female with age 60 ± 10 years and diabetes duration 45 ± 11 years. Twelve (80%) had received islet transplants alone and three (20%) islet after kidney transplants. Questionnaires were completed at a mean of 6.2 years following first islet transplant.

Ten (67%) participants were using multiple dose insulin therapy with the remaining five (33%) on continuous subcutaneous insulin infusion pumps. Eight (53%) participants were using continuous glucose monitoring and the remaining seven (47%) flash glucose monitoring. Pre-transplant total daily insulin dose in the cohort as a whole was 0.47 ± 0.19 units/kg.

At the time of cross-sectional assessment, five (33%) participants’ transplants were classified as having “Good” function, four (27%) had “Marginal” function and six (40%) had “Failed.” “Good” function required absence of severe hypoglycaemia and HbA1c <7.0% (53 mmol/mol) [6]. C-peptide increase compared to baseline was defined as stimulated C-peptide >50 pmol/L, as all had mixed meal tolerance test 90 min values below this cut-off at baseline. Those in the ‘Good’ function group had reductions in total daily insulin dose of >50% with the exception of one participant with current insulin dose of 0.15 units/kg where pre-transplant insulin dose was not available and another with very low pre-transplant insulin dose (0.34 units/kg) reduced by only 44% to 0.19 units/kg at post-transplant assessment. “Marginal” function was defined as HbA1c ≥7.0 (53 mmol/mol)), severe hypoglycaemia with less than baseline frequency, insulin requirement ≥50% of baseline and C-peptide level greater than baseline [4]. Those who had stimulated C-peptide <50 pmol/L were defined as having a graft that had “Failed.”

Age, duration of diabetes and number of transplants were comparable in all groups (Table 1). Time since first transplant tended to be longest in those with graft failure. Reduction in severe hypoglycaemia event rate of >90% was sustained following islet transplantation even in those with graft failure. Nevertheless, only the group with “Good” function had no individuals experiencing severe hypoglycaemia at the time of assessment (Table 1). There was a trend towards incrementally higher HbA1c with worsening graft status.

**TABLE 1:** Cross-sectional metabolic status of transplant recipients classified, by Igls graft status.

	Islet graft function			*p*-value
	Good	Marginal	Failed	
Number	5 (33%)	4 (27%)	6 (40%)	—
Age (years)	62.0 ± 16.6	61.3 ± 5.3	57.0 ± 4.1	0.696
Duration of diabetes (years)	50.0 ± 16.8	38.8 ± 7.8	44.7 ± 4.5	0.389
Female	4 (80%)	3 (75%)	5 (83%)	1.000
Islet transplant alone	4 (80%)	4 (100%)	4 (67%)	0.736
Number of islet transplants	2 (2.2)	2 (1.2)	2 (2.2)	0.857
Insulin independence achieved	3 (60%)	1 (25%)	1 (17%)	0.397
Time since first transplant (months)	59.0 ± 35.3	59.8 ± 44.7	97.7 ± 30.3	0.173
CSII	0 (0%)	2 (50%)	3 (50%)	0.201
CGM	2 (40%)	3 (75%)	3 (50%)	0.674
Severe hypoglycaemia:				
Frequency per year	0.0 ± 0.0	1.5 ± 1.0	1.7 ± 2.3	0.093
Participants per year	0 (0%)	3 (75%)	3 (50%)	0.069
HbA1c (mmol/mol)	49 ± 4	56 ± 5	69 ± 25	0.389
Daily insulin dose (units/kg)	0.15 ± 0.03	0.15 ± 0.09	0.53 ± 0.15	<0.001
Percentage reduction in daily insulin dose (compared with pre-transplant)	54.1%	49.0%	3.7%	0.033^+^
C-peptide (pmol/L)	658 ± 372	218 ± 59	0.0 ± 0.0	0.002
Concomitant glucose (mmol/L)	6.8 [6.8, 8.2]	8.65 [6.4, 11.3]	7.3 [5.2, 9.4]	0.639
C-peptide: glucose ratio (nmol/L:mmol/L)	0.055 [0.045, 0.072]	0.028 [0.025, 0.029]	0.001 [0.000, 0.001]	0.002

Data are n (%); mean ± SD or median (Q1,3). Means were compared by one way ANOVA and medians by Kruskal-Wallis test. Categorical data were compared by Fisher’s exact test. CSII, continuous subcutaneous insulin infusion; CGM, continuous glucose monitoring; HbA1c, glycated haemoglobin.

Insulin dose was significantly lower in both groups with graft function, compared to the graft failure group, with comparable dose (∼50% of baseline requirements) in individuals with Igls “Marginal” and those with “Good” function. A period of insulin independence was achieved in 60% of those with current “Good” function but in ≤25% within the other two groups.

Random C-peptide was significantly lower in the “Marginal” compared with the ‘Good’ function group with C-peptide/glucose ratio falling incrementally with worsening graft function.

All participants with ongoing graft function were on a comparable immunosuppression regimen (tacrolimus with/without mycophenolate mofetil). Three (50%) of those classified as ‘failed’ were no longer taking immunosuppression. Two remained on immunosuppression for a functional renal transplant and one remained on low dose tacrolimus alone immunosuppression on the active waiting list for islet retransplantation.

Although only 15 individuals (71% of those who fulfilled inclusion criteria) consented to participate and completed questionnaires, they appeared representative of the overall cohort. The remaining 6 exogenous insulin-requiring islet transplant recipients in this single site cross-sectional study included a comparable distribution of those with “good,” “marginal” and “failed” function. All had received islet transplants alone. Age and duration of diabetes were comparable to the study participants and the majority were female.

### Self-Reported Hypoglycaemia Awareness and Experience

Across all three insulin-requiring Igls groups, most recipients self-reported unresolved impairment in hypoglycaemia awareness without significant differences in Gold or HypoA-Q “Impaired Awareness” scores in those with “Good,” “Marginal” or “Failed” graft function (Table 2).

**TABLE 2:** Hypoglycaemic awareness, by Igls criteria.

	Islet graft function			*p*-value
	Good	Marginal	Failed	
Awareness of hypoglycaemia: Gold Score	6 [6, 6]	4 [1, 7]	6 [3.5, 7]	0.849
Impaired awareness of hypoglycaemia: Gold Score ≥4	4 (80%)	2 (50%)	4 (67%)	0.800
Hypoglycaemia Awareness: HypoA-Q				
Impaired Awareness (/20)	10 ± 4	10 ± 5	9 ± 4 (*n* = 5)	0.862
Symptom Frequency (/30)	10.8 ± 4.2	23.7 ± 7.8 (*n* = 3)	19.4 ± 2.7 (*n* = 5)	0.008
Symptom Level (/18)	16 [13, 17]	18 [17, 18]	13 [12, 17] (*n* = 5)	0.152

Data are n (%); mean ± SD or median (Q1,3). Means were compared by one way ANOVA and medians by Kruskal-Wallis test. Categorical data were compared by Fisher’s exact test. When data incomplete, number with available data denoted by n number in parentheses. IAH, Impaired Awareness of Hypoglycaemia.

In addition to HypoA-Q hypoglycaemia awareness scoring, novel analysis of HypoA-Q “Symptom Frequency” (Supplementary Table S2) and “Symptom Level” subscales (Supplementary Table S3) was undertaken. The Symptom Frequency question was scored in two parts. In the first, participants indicated whether in the past month they had experienced blood glucose readings in the following ranges: 3.5–3.9 mmol/L (1 point); 3.0–3.4 mmol/L (2 points); 2.5–2.9 mmol/L (3 points); <2.5 mmol/L (4 points). In Part 2, participants were asked how often symptoms are experienced if they encounter glucose levels within each of these ranges: never (5 points); rarely (4 points); sometimes (3 points); often (2 points); always (1 point). Higher scores indicate more experience of more profound biochemical hypoglycaemia with less frequent symptoms.

Analysis of the HypoA-Q “Symptom Frequency” subscale showed 100% with “Marginal” graft function and 80% with “Failed” function reported experiencing glucose <2.5 mmol/L within the last month, whereas none of those with “Good” graft function reported levels in this range. When hypoglycaemia was experienced, participants with “Good” graft function experienced symptoms more often than those with “Marginal” (*p* = 0.01) or “Failed” (*p* = 0.039) function (Table 2).

The HypoA-Q “Symptom Level” subscale (question 6) asks the participant “how low does your blood glucose usually need to be before you feel” one or more symptoms, clustered into: autonomic, neuroglycopaenic and non-specific. Higher scores are allocated to lower glucose thresholds with a maximum score of 18 (Supplementary Table S3). All groups scored highly on HypoA-Q “Symptom Level” subscale (Table 2), consistent with unresolved impairment of awareness evidenced by absence of symptoms regardless of how low glucose falls or, at least, requirement for glucose levels below those required for normal cognitive functioning before any symptoms are experienced.

### Fears, Attitudes and Behaviours Around Hypo- and Hyperglycaemia

Worry about hypoglycaemia appeared low only in those with “Good” graft function (Table 3), although differences between groups did not reach statistical significance. Worry related to high glucose levels (measured by the Hyperglycaemia Avoidance Scale; HAS) appeared to increase incrementally with worsening graft function category, although again differences were not significant.

**TABLE 3:** Worries, behaviours and attitudes to hyper- and hypoglycaemia, by Igls graft status.

	Islet graft function			*p*-value
	Good	Marginal	Failed	
Fear of hypoglycaemia: HFS-II				
Behaviour	30 [28, 33]	51 [43, 52]	28 [24, 36] (*n* = 4)	0.303
Worry	4 [3, 28]	62 [49, 69]	57 [17, 64] (*n* = 5)	0.067
Total	33 [29, 61]	113 [95, 119]	63 [42, 93] (*n* = 4)	0.082
Hyperglycaemia avoidance: HAS				
Immediate Action	10 [8, 11]	9 [7, 10]	10 [4, 12]	0.944
Worry	14 [14, 27]	22 [22, 28]	32 [22, 33] (*n* = 5)	0.307
Low Blood Glucose Preference	4 [3, 5]	4 [2, 6]	7 [5, 13]	0.207
Avoid Extremes	4 [1, 5]	7 [4, 12]	6 [4, 9] (*n* = 5)	0.372
Attitudes to Awareness: A2A				
Asymptomatic hypoglycaemia normalised	0 [0, 1]	1 [1, 1]	0 [0, 4] (*n* = 5)	0.755
Hypoglycaemia concerns minimised	1 [1, 2]	1 [1, 2]	0 [0, 1] (*n* = 5)	0.246
Hyperglycaemia avoidance prioritised	5 [3, 5]	3 [3, 4]	4 [3, 6] (*n* = 5)	0.899

Data are median (Q1,Q3). Medians were compared by Kruskal-Wallis test. When data incomplete, number with available data denoted by n number in parentheses. HFS, Hypoglycaemia Fear Survey; HAS, Hyperglycaemia Avoidance Scale; A2A, Attitudes to Hypoglycaemia.

Drive to take immediate action to reduce high blood glucose (measured by the HAS) and prioritisation of hyperglycaemia avoidance (measured by the Attitudes to Awareness questionnaire; A2A) were scored highly in all groups suggesting underlying behavioural preferences which are not influenced by islet graft function. Low blood glucose preference (HAS) and asymptomatic hypoglycaemia normalised/hypoglycaemia concerns minimised (A2A) were scored relatively low by participants in all three Igls groups, consistent with the approach within the UK islet transplant programme of only listing individuals who recognise concerns regarding dangerous hypoglycaemia risk as their primary motivator for proceeding with transplantation despite the need for life-long immunosuppression.

### Diabetes Distress and General Anxiety/Depressive Symptoms

Median PAID scores of those in both the “Marginal” and “Failed” graft function groups indicated they were experiencing elevated diabetes distress (PAID score >40), contrasting with those with “Good” graft function who reported lower median PAID scores (Table 4). Assessed using the Type 1 Diabetes Distress Scale (T1DDS), diabetes distress scores were highest in the group with “Marginal” graft function who reported “moderate/high distress” in all domains, incrementally lower in the group with “Failed” function, but were within the little/no distress range for 5 of 7 domains in those with “Good” islet graft function (Table 4). Highest scores in those with “Marginal” function reached statistical significance for “physician distress.”

**TABLE 4:** Diabetes distress and general anxiety/depressive symptoms, by Igls graft status.

	Islet graft function			p-value
	Good	Marginal	Failed	
Diabetes distress: PAID	23 ± 26	44 ± 29 Severe distress	41 ± 17 (*n* = 5) Severe distress	0.211
Diabetes distress: T1DDS				
Powerlessness	2.1 ± 0.9 Moderate	3.6 ± 1.7 High	2.8 ± 1.2 (*n* = 5) Moderate	0.272
Management distress	1.0 [1.0, 1.3] Little/none	1.5 [1.2, 2.5] Moderate	1.5 [1.5, 1.5] (*n* = 5) Little/none	0.136
Hypoglycaemia distress	2.2 ± 1.3 Moderate	4.0 ± 1.5 High	3.0 ± 1.4 (*n* = 5) High	0.188
Negative social perceptions	1.6 ± 1.7 Little/none	2.8 ± 2.4 Moderate	2.4 ± 1.5 (*n* = 5) Moderate	0.649
Eating distress	1.3 [1.0, 1.3] Little/none	1.3 [1.0, 2.8] Moderate	2.0 [2.0, 2.3] (*n* = 5) Moderate	0.173
Physician distress	1.0 [1.0, 1.0] Little/none	2.5 [1.8, 3.1] Moderate	1.0 [1.0, 1.0] (*n* = 5) Little/none	0.032
Friend/family distress	1.5 [1.0, 1.5] Little/none	2.8 [2.1, 3.1] Moderate	1.0 [1.0, 2.3] (*n* = 5) Little/none	0.361
Anxiety symptoms:				
HADS - A	8 ± 4 Mild	13 ± 6 (*n* = 3) Moderate	9 ± 2 Mild	0.216
GAD-7	6 [3, 11] Mild	21 [17, 21] (*n* = 3) Severe	6 [6,6] (*n* = 5) Mild	0.079
Depressive symptoms:				
HADS - D	6 [4, 8] Normal	11 [8.5, 11] (*n* = 3) Moderate	7 [6, 8] Normal	0.548
PHQ-9	7 [5, 8] Mild	27 [19, 27] (*n* = 3) Severe	10 [7, 11] (*n* = 5) Moderate	0.077

Data are mean ± SD or median (Q1,3). Means were compared by one way ANOVA, and medians by Kruskal-Wallis test. When data incomplete, number with available data denoted by n number in parentheses. PAID, Problem Areas in Diabetes; T1DDS, Type 1 Diabetes Distress Scale; HADS - A, Hospital Anxiety and Depression scale–Anxiety subscale; HADS-D, Hospital Anxiety and Depression scale–Depression subscale; PHQ-9, Patient Health Questionnaire-9.

Similarly, self-reported generalised anxiety and depressive symptom scores were highest, and in the “severe range” in those with “Marginal” graft function (Table 4), and lowest among those with “Good” function.

### Participant Experience of PROMs

Feedback on individual questionnaires and overall usefulness/burden of questionnaire completion was provided by 11 particiopants and was largely positive (Table 5). All were perceived as valuable with no consistent strong preference for one questionnaire over another. The importance of reviewing questionnaire responses with those completing them, acknowledging issues arising and acting on these where appropriate was emphasised, with one participant stating: “I would like it to be compared to other questionnaire answers and if anything was significantly different for this to be addressed. Hopefully information gathered will help to work on giving diabetics a better way of managing their every day lives.”

**TABLE 5:** Participant feedback on questionnaires.

Questionnaires	Feedback
Overall pack	“Questionnaire was fine”
	“Overall they were ok, not time consuming”
	“A lot were repetitive, parts I do not understand due to terminology”
	“Time consuming”
	“I thought it covered everything relevant”
	“Absolutely fine and went through lots of helpful information via the questions asked”
Were the questions within the questionnaires relevant to you? If so, which	“Very relevant”
	“Yes”
	“Most of the questions”
	“Yes very relevant, well most of them”
Do you feel that the pack addresses all aspects of living with diabetes and/or following islet transplantation?	“How I felt after transplant and how it improved my quality of life”
	“Yes”
	“I think yes the questions covered everything”
	“Could consider a part about side effects of transplant/transplantation medications and how that affects your diabetes”
	“I think it is extremely difficult to remove a questionnaire relating specifically to circumstances transplant history and feelings, everyone is different”
Gold Score	“Excellent”
HypoA-Q	“Hard to think back 6 months”
	“I liked the way the signs of a hypo were so accurate”
	“I liked that it was thorough”
	“Very good it makes one think about how they might manage hypo-awareness”
HFS-II	“That is first a question for individuals however I think every diabetic is scared or worried about hypos”
A2A	“Should add do people understand what is going on with you a lot of the time people think you are drunk or having a fit”
	“Made me realise I act on my sensor telling me that sugar is low, not very aware and not concerned as I do not go below 3”
	“This is interesting for me because I have always managed my diabetic control in a way that I run a low blood sugar. I am aware of the problems associated with this way of doing things but I do not want to have high BG’s.”
HAS vs. A2A	“Attitudes to awareness of hypos was more about how you feel”
	“I could not answer some of the questions”
	“Covered feelings I have when my blood sugars are high and actions I have taken”
	“Personally speaking ‘high blood glucose’ is a real worry for all the outlined listed issues”
PAID	“Worries a lot about not having long to live. I think I am going to die before I am 66. I also think I am going to be ill, very ill and needing to have dialysis, so doing the questionnaire was both good and bad. I am ok right now.”
T1-DDS	“A bit complicated and hard to think back over the last month”
	“All very thorough and included different aspects of life”
PAID vs. T1-DDS	“More options to explain how things affect you and make you feel in the T1-DDS”
	“I am aware of the problems associated with diabetes”
HADS	“It is good that you recognised anxiety and depression as part of diabetic life because a lot of the time it is ignored”
	“I do suffer from anxiety and I think that sometimes my diabetes plays a part in this feeling, the constant worry and concern about being well, having hypos etc. is always going to be part of this”
HADS vs. GAD-7/PHQ-9	“Both are big problem areas with diabetes”
	“Not really have a preference”
	“Extremely similar”

## Discussion

In this study, we set out to determine whether “Good” and “Marginal” islet graft function (defined by the EPITA/IPITA consensus) were able to differentiate person-reported experience and outcomes. Fifteen adults with (pre-transplant) C-peptide negative type 1 diabetes were studied cross-sectionally at a mean of 6 years following their first islet transplant at a single centre. All required at least low dose insulin replacement but were continuing to benefit from significantly reduced severe hypoglycaemia. Despite maintained biomedical benefit, person-reported measures of health status revealed significant concerns in those with “Marginal” graft function including persistent fear regarding hypoglycaemia, diabetes distress and overall anxiety symptoms in contrast to those with “Good” function. This provides validation of the Igls criteria in meaningfully defining overall clinical outcomes through a simple biomedical scoring system. In addition, the current study provides evidence of unmet needs in those with “Marginal” function justifying further intervention, including re-transplantation.

Recurrent life-threatening severe hypoglycaemia remains the primary indication for deceased donor islet transplant, both within the NHS-adopted integrated UK programme and more widely. We and others have previously reported that significant biochemical hypoglycaemia (glucose <3 mmol/L) can be successfully avoided even in those with relatively low levels of restored C-peptide secretion following islet transplant [17], in keeping with the ongoing reduction in severe hypoglycaemia in all groups in the current study. Nevertheless, 75% with “Marginal” and 50% with “Failed” graft function had experienced at least one episode of severe hypoglycaemia over the preceding year. Following careful assessment of person-centred outcomes in a cohort of islet transplant recipients in Edmonton, it has been proposed that meal tolerance test stimulated C-peptide of >680 pmol/L is required for freedom from serious, clinically important hypoglycaemia [18]. Although MTTs were not undertaken in this cross-sectional study, it is clear from random C-peptide values that only those classified as Igls “Good” had sustained this level of graft function.

Classification of graft function as “Marginal” in the presence of any severe hypoglycaemic events has been widely accepted. Exclusion of all with HbA1c >7% from the “Good” function category has been more contentious, even though HbA1c <7% was agreed by NICE as a key performance indicator for reimbursed islet transplantation and formed part of the primary outcome measure for the US Phase 3 trials towards islet transplant licensing [3, 19]. The current analysis supports this cut-off as a meaningful marker for graft impairment sufficient to negatively impact recipient confidence and well-being. Forty-two percent (3 of 7) of those with HbA1c >7% versus 25% (2 of 8) of those with HbA1c <7% had experienced severe hypoglycaemia over the preceding 12 months. In our continuous glucose monitoring analysis of an earlier cohort [17], HbA1c post-islet transplant was shown to be intrinsically related to graft function, regardless of exogenous insulin dose adjustment and individual therapeutic goals, with mean HbA1c 6.9% in those with stimulated C-peptide in the range 500–1,000 pmol/L, mirroring data in the current cross-sectional study. In a recent analysis of 677 islet transplant alone recipients within the Clinical Islet Transplant Registry, C-peptide was highly associated with concurrent metabolic status, with mixed meal tolerance test-stimulated C-peptide-to-glucose ratio (CPGR) having optimal predictive value [20]. Optimal CPGR cut-points for predicting absence of severe hypoglycaemia and HbA1c <7% were 0.044 nmol/mmol (fasting) and 0.071 nmol/mmol (stimulated). This is in keeping with the mean random CPGR of 0.055 nmol/mmol in the Igls “Good” group in the current study.

The evidence that optimal primary graft function predicts long term graft function is now incontrovertible [21]. In the current study, 60% of those with sustained “Good” function at a mean of 6.2 years post-first islet transplant had attained a period of insulin independence, whereas only 25% of those with “Marginal” function had experienced this.

Previous studies have confirmed the potential to restore counter-regulatory response and improve symptomatic response to hypoglycaemia following optimal islet engraftment [22]. In the current study, however, at least 50% of participants continued to self-report impaired awareness of hypoglycaemia regardless of level of graft function. Although no differences between groups were manifest using validated IAH scores, a novel analysis of HypoA-Q “Symptom Frequency” and “Symptom Level” subscales (Supplementary Table S2) showed that only participants with “Good” function were not experiencing glucose levels <2.5 mmol/L and that, when this was experienced, it was associated with more frequent symptoms when glucose was 2.5–3.9 mmol/L). Although two scores were used, the validated Clarke survey was not employed and may be a more sensitive instrument for differentiating degrees of impaired awareness [23, 24]. We did not include the Clarke survey given the inclusion of questions within the score around hypoglycaemia severity as well as those specifically assessing awareness. Continuous glucose monitoring metrics were also not included. These may have revealed biochemical hypoglycaemia exposure sustaining IAH even in those with “good” function, whereas absolute hypoglycaemia avoidance would be envisaged in insulin independent recipients with “optimal” function.

The factors associated with incomplete recovery of awareness even following successful biochemical hypoglycaemia avoidance remain unclear [25]. We hypothesise that cognitive and physical frailty in those being considered for islet transplantation may be contributory factors given mean age 60 years with diabetes duration 45 years in the current cohort. This study demonstrates the value of using the long-form of HypoA-Q, incorporating two additional questions to more fully characterise symptomatic response to hypoglycaemia, enabling discrimination between groups with differing C-peptide levels.

There have been relatively few previous studies in islet transplant recipients evaluating wider person-reported impact using validated questionnaires, and PROMs have not previously been utilised to validate Igls criteria [26–29]. Reduced fear of hypoglycaemia has been reported in a number of cohorts following successful islet transplant [28, 29]. The current analysis suggests that this requires “Good” graft function, with highest overall HFS score in recipients with “Marginal” function.

Attitudes and behaviours related to high glucose levels have not previously been reported in islet transplantation and were undertaken using the validated Hyperglycaemia Avoidance Scale (HAS) [12] and Attitudes to Awareness questionnaire [15]. High scores for “hyperglycaemia avoidance prioritised” and “immediate action for hyperglycaemia” were reported irrespective of islet transplant function reflecting underlying cognitions contributing to increased hypoglycaemia tendency in this cohort. These values are comparable to those at baseline in the HypoCOMPaSS multicentre trial of UK participants with long-standing type 1 diabetes and impaired awareness of hypoglycaemia [30], suggesting that islet transplantation does not impact these long-established attitudinal beliefs. Worry regarding hyperglycaemia appears to be attenuated in islet transplant recipients with “Good” graft function mirroring reduced fear of hypoglycaemia.

Islet transplant recipients in all groups continued to report a strong preference for low versus high blood glucose levels but neither “minimisation of concerns regarding hypoglycaemia” nor “normalisation of asymptomatic low blood glucose levels” were endorsed strongly in this cohort. This likely reflects the screening process within our programme, which requires each individual to be clear that dangerous hypoglycaemia is their over-riding concern, justifying progression to beta-cell replacement therapy despite the associated risks.

Worry about and avoidance of circumstances that might lead to high glucose (constituting the HAS Avoid Extremes subscale) was scored particularly highly in islet transplant recipients, compared with those in the HypoCOMPaSS trial [30], potentially evidencing a strong desire to minimise glucotoxic stress to the transplanted cells.

Reduced diabetes distress following successful islet transplantation has been reported previously [29]. In this study, T1DDS scores were highest in the “Marginal” graft function group, suggesting that having biomedical evidence of persistent graft function with a parallel increase in HbA1c and risk of severe hypoglycaemia may be particularly distressing, even compared to those who have lost function all together. Scores for “physician distress” were significantly higher in those with “Marginal” function. This sub-scale includes the “feeling that I do not get help I really need from my diabetes doctor about managing diabetes,” This loss of confidence may be at least partially driven by healthcare professional assurances that the graft is still functioning, without fully acknowledging recipients’ recognition that glucose unpredictability and hypoglycaemia risk has recurred associated with diabetes distress. Global anxiety and depressive symptoms (GAD-7 and PHQ-9) scores were in the severe range for those with “Marginal” function.

Significant anxiety and low mood were, however, reported by all groups. This may be attributable to their high-risk status and requirement for prolonged “shielding” during the COVID-19 pandemic, but nevertheless confirms that successful islet transplantation (certainly in the absence of predictable, sustained insulin independence) does not completely allay ongoing concerns, given the need for ongoing close monitoring and the potential risks of concomitant immunosuppression.

It is possible that high anxiety and depression scores in those with “marginal function” were contributed to by continued immunosuppression despite marginal perceived benefits. We do not, however, advocate immunosuppression withdrawal in this C-peptide positive group where biomedically meaningful graft function is maintained and given the associated risk of alloantibody sensitisation [31]. In contrast those with failed function were only continuing to take immunosuppression to maintain a functional renal graft or in preparation for further islet transplantation.

As currently configured, Igls “Good” status is dependent on meeting criteria for all four included parameters (C-peptide; severe hypoglycaemia; HbA1c; and insulin dose). We strongly endorse the need for graft function in all islet transplant recipients to be primarily evidenced by robust demonstration of higher C-peptide compared to pre-transplantation. We believe that the current analysis provides strong supportive evidence for classifying all with HbA1c >7% as “Marginal.” While understanding that lower HbA1c may be attainable by higher dose exogenous insulin, we conclude that there is now sufficient evidence for an intrinsic impact of C-peptide on composite hypoglycaemia/HbA1c outcomes [17, 18]. Moreover, exogenous insulin needs may be influenced by concomitant oral glucose-lowering agents. We thus suggest that the absolute requirement for >50% reduction in insulin dose post-islet transplant is removed from the criteria justifying designation of islet transplant function as “Good” as proposed at the International Pancreas and Islet Trasnplant Consortium 2021 Igls criteria symposium and further supported by the current PROM analysis.

Limitations of this study include relatively small numbers of participants within a single centre and the cross-sectional design without inclusion of pre-transplant PROMs. Further studies will be important to confirm the current findings in larger prospective cohorts including insulin independent recipients with “optimal” function.

A key strength is the comprehensive assessment of PROMS, and the robust process that we undertook to select appropriate validated measures and to confirm face-validity and utility for implementation in people undergoing islet transplantation. Despite the effort required in completion, participants fed-back positively regarding the value of collecting and reviewing PROM data in parallel with biomedical outcomes. An unmet need to assess anxiety and depression was clearly identified.

For holistic assessment following islet transplantation, we recommend a “minimum dataset” PROM pack quantifying hypoglycaemia awareness, hypoglycaemia fear, diabetes distress, anxiety and depression. Suggested specific measures are Gold score, HFS-II, PAID and HADS.

In addition to providing person-reported outcome validation of cut-offs selected by the healthcare professional community for Igls islet transplant function criteria, this study has confirmed the importance of collecting, reporting and responding to more holistic assessment of wider factors necessary for overall well-being and truly optimal outcomes, using a standardised PROM questionnaire pack in parallel with interval biomedical data collection.

## Data Availability

The original contributions presented in the study are included in the article/Supplementary Material, further inquiries can be directed to the corresponding author.
