# Supplementary material for: Validation of Igls Criteria for Islet Transplant Functional Status Using Person-Reported Outcome Measures in a Cross-Sectional Study
Source: Transpl Int. 2023 Sep 26;36:11659. doi: 10.3389/ti.2023.11659 (PMC10563803; doi:10.3389/ti.2023.11659)
Supplement: Supplementary file 1 [file DataSheet1.docx]

Supplementary Material

Validation of Igls criteria for islet transplant functional status using person-reported outcome measures in a cross-sectional study

Zoe Bond^1^, Saffron Malik^1^, Ayat Bashir^1^, Rachel Stocker^2^, Jocelyn Buckingham^3^, Jane Speight^4 5^, James AM Shaw^1^

^1^Translational and Clinical Research Institute, Newcastle University, Newcastle upon Tyne, U.K.
^2^School of Biomedical, Nutritional, and Sport Sciences, Newcastle University, Newcastle upon Tyne, U.K.

^3^Newcastle upon Tyne Hospitals NHS Foundation Trust, Newcastle upon Tyne, U.K.

^4^School of Psychology, Institute for Health Transformation, Deakin University, Geelong, Victoria, Australia.

^5^The Australian Centre for Behavioural Research in Diabetes, Diabetes Victoria, Carlton, Victoria, Australia.

*** Correspondence:** Corresponding Author: jim.shaw@ncl.ac.uk

# Supplementary Figures and Tables

## Table 1: Summary of questionnaires completed by participants in this study.

| **The Gold Score** (13) | Assesses self-reported awareness of hypoglycaemia. The score comprises a 7-point Likert scale relating to the question ‘Do you know when your hypos are commencing?’ with 1 = ‘always aware’ and 7 = ‘never aware’. A score of ≥4 indicates IAH. |
| --- | --- |
| **The HypoA-Q** (16) | Assesses hypoglycaemia awareness and the impact of this on frequency of associated symptoms and glucose levels at which they are experienced. Scored using 3 subscales: ‘Impaired awareness’, ‘Symptom level’ and ‘Symptom frequency’. |
| **The Hypoglycaemia Fear-Survey-II (HFS-II)** (11) | Comprises behaviour (HFS-B) and worry (HFS-W) subscales, assessing behaviours potentially driven by fear of low glucose levels and worry associated with this, considering the preceding 6 months. There are 33 items, rated on a scale of 0 = ‘never’ to 4 = ‘almost always’. Higher scores indicate increased hypoglycaemia avoidance behaviours and greater worry regarding hypoglycaemia. |
| **The Hyperglycaemia Avoidance Scale (HAS)** (12) | Assesses avoidance of hyperglycaemia and how this may in turn affect frequency and severity of hypoglycaemia. Scored using 4 subscales: ‘Immediate action’, ‘Worry’, ‘Low BG preference’, and ‘Avoid extremes’. |
| **The Attitudes to Awareness of Hypoglycaemia (A2A)**(7) | Two-part questionnaire assessing understanding of IAH, motivation to regain awareness and whether or not a person agrees with a range of health beliefs. The health belief catergories are: ‘normalise hypoglycaemia unawareness’, ‘underestimate hypoglycaemia unawareness’, ‘avoid sick role’, ‘overestimate high glucose’. |
| **The Problem Areas in Diabetes (PAID)** (8) | Designed to help recognise diabetes-related stress including general emotional distress, fear of hypoglycaemia (FOH), eating disorder, diabetic complications and adherence to regimes. |
| **The Type 1 Diabetes Distress Score (T1-DDS)**(15) | 28-item measure, made up of 7 subscales addressing major sources of distress for people with diabetes. Overall average scores are calculated, as well as an average score for each subscale. Reviewing the individual subscale scores allows causes of high levels of distress for each patient to be identified. |
| **The Hospital Anxiety and Depression Scale (HADS)** (9) | Contains 7 questions addressing general anxiety and 7 questions addressing general depression, evaluating anxiety/depressive symptoms experienced over the previous week. Determines both the presence and severity of symptoms. |
| **The GAD-7** (14) | Measure for evaluating the presence of Generalised Anxiety Disorder (GAD) in clinical settings, as well as assessing the severity. The points of 5, 10 and 15 represent mild, moderate and severe levels of anxiety respectively. |
| **The PHQ-9** (10) | Scores for 9 DSM-IV criteria, making it a reliable clinical measure of depression. |

## Table 2: Scoring system for the novel analysis of HypoA-Q: symptom frequency subscale

| **Symptom frequency subscale:**  Total score /30 | **Part 1:**  In the past month, have you had blood glucose readings (in mmol/l)… | | **Part 2:**  **If yes**, how often did you have hypo symptoms? |
| --- | --- | --- | --- |
| Question 5a | ‘3.5-3.9 mmol/l’ | Yes = 1  No = 0  Don’t know = 0 | Never = 5  Rarely = 4  Sometimes = 3  Often = 2  Always = 1 |
| Question 5b | ‘3.0-3.4 mmol/l’ | Yes = 2  No = 0  Don’t know = 0 | Never = 5  Rarely = 4  Sometimes = 3  Often = 2  Always = 1 |
| Question 5c | ‘2.5-2.9 mmol/l’ | Yes = 3  No = 0  Don’t know = 0 | Never = 5  Rarely = 4  Sometimes = 3  Often = 2  Always = 1 |
| Question 5d | ‘Less than 2.5 mmol/l’ | Yes = 4  No = 0  Don’t know = 0 | Never = 5  Rarely = 4  Sometimes = 3  Often = 2  Always = 1 |

## Table 3: Scoring system for the novel analysis of HypoA-Q: symptom level subscale

| **Symptom level subscale:** | How low does your blood glucose usually need to be before you feel any of the following symptoms?  **Total score /18** | |
| --- | --- | --- |
| Question 6a | ‘Trembling, shakiness, pounding heart, warmth, sweating, hunger’ | 4.0mmol/l or above = 1  3.5-3.9mmol/l = 2  3.0-3.4mmol/l = 3  2.5-2.9mmol/l = 4  <2.5mmol/l = 5  ‘I do not have these symptoms’ = 6 |
| Question 6b | ‘Weakness, lack of coordination, confusion, dizziness, inability to concentrate, difficulty speaking, blurred vision, drowsiness, tiredness, irritability, odd behaviour’ | 4.0mmol/l or above = 1  3.5-3.9mmol/l = 2  3.0-3.4mmol/l = 3  2.5-2.9mmol/l = 4  <2.5mmol/l = 5  ‘I do not have these symptoms’ = 6 |
| Question 6c | ‘Nausea, tingling, headache’ | 4.0mmol/l or above = 1  3.5-3.9mmol/l = 2  3.0-3.4mmol/l = 3  2.5-2.9mmol/l = 4  <2.5mmol/l = 5  ‘I do not have these symptoms’ = 6 |

**
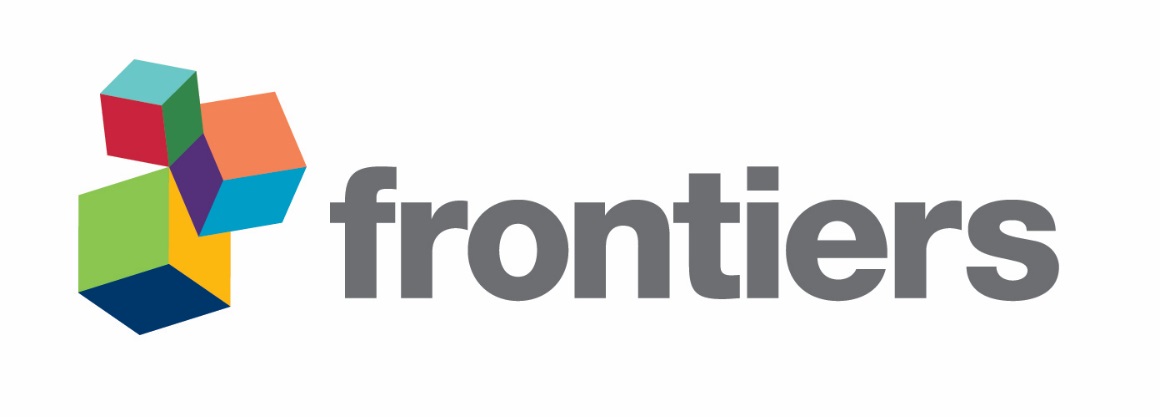
**
